# *Primulina cardaminifolia* (Gesneriaceae), a rare new species from limestone areas in Guangxi, China

**DOI:** 10.1186/1999-3110-54-19

**Published:** 2013-08-27

**Authors:** Wei-Bin Xu, Yan Liu, Yoshiko Kono, Hsuan Chang, Ching-I Peng, Kuo-Fang Chung

**Affiliations:** 1 Guangxi Institute of Botany, Guangxi Zhuangzu Autonomous Region and the Chinese Academy of Sciences, Guilin, 541006 China; 2 Herbarium (HAST), Biodiversity Research Center, Academia Sinica, Nangang, Taipei, 115 Taiwan; 3 School of Forestry and Resource Conservation, National Taiwan University, Daan, Taipei, 106 Taiwan

**Keywords:** Chromosome number, Flora of China, Homoploid hybrid, Sino-Vietnamese limestone karst, Molecular taxonomy, *Primulina pinnata*

## Abstract

**Background:**

*Primulina cardaminifolia* Yan Liu & W.B. Xu (Gesneriaceae), a distinct new species with imparipinnate leaves, is described and illustrated from a limestone valley in Guangxi Zhuangzu Autonomous Region, China. To assure its generic placement and phylogenetic affinity, phylogenetic analyses were performed using DNA sequences of nuclear ITS and chloroplast *trnL-F* intron spacer region. Additionally, somatic chromosome number was counted and pollen stainability was tested.

**Results:**

Phylogenetic analyses support its placement in *Primulina*; however, two phylogenetically distinct ITS sequence types were detected, suggesting a probable hybrid origin. Its pollen stainability is 100% and its chromosome number, 2*n* = 36, is congruent with all known counts of diploid species of the genus.

**Conclusion:**

All available data support the recognition of the new species *Primulina cardaminifolia* and suggest that it could have derived from homoploid hybrid speciation. Color plates, line drawings and a distribution map are provided to aid in identification.

**Electronic supplementary material:**

The online version of this article (doi:10.1186/1999-3110-54-19) contains supplementary material, which is available to authorized users.

## Background

Recent progress in molecular phylogenetic studies of the Old World Gesneriaceae has foreseen the restructuring of the highly heterogeneous *Chirita* D. Don (Li and Wang 2007; Möller et al. 2009; Möller et al. 2011). However it was unexpected that *Chirita* has been forsaken (Wang et al. 2011; Weber et al. 2011) and the formerly monotypic *Primulina* Hance recircumscribed and expanded to include *Chirita* sect. *Gibbosaccus* C.B. Clarke, *Chiritopsis* W.T. Wang, and two species of *Wentsaiboea* D. Fang & D.H. Qin (Wang et al. 2011; Weber et al. 2011). With over 130 species transferred to (Wang et al. 2011; Weber et al. 2011; Xu et al. 2012b) and more than ten species newly described (Liu et al. 2011; Hong et al. 2012; Huang et al. 2012; Li et al. 2012; Wen et al. [Bibr CR26][Bibr CR27][Bibr CR28]; Wu et al. [Bibr CR29][Bibr CR30]; Xu et al. 2012a; Chung et al. 2013), *Primulina* has now become one of the largest genera of the Old World Didymocarpoid Gesneriaceae. Under this new delimitation, *Primulina* is essentially a calciphilous genus distributed in southern China and adjacent northern Vietnam, with the major center of diversity in the limestone karsts of Guangxi Zhuangzu Autonomous Region (Wang et al. 1998; Li and Wang 2004; Hou et al. 2010; Wei 2010; Weber et al. 2011; Xu et al. [Bibr CR31][Bibr CR32]).

Although the new generic circumscription reflects better the evolutionary relationships and ecological preferences of the genus, diagnostic characters of *Primulina*, such as perennial habit and acaulescent rosette (2011) and the basic chromosome number, *x* = 18 (Christie et al. 2012; Liu et al. 2012), are not exclusive to the genus. The lack of strong morphological synapomorphies to distinguish it from related genera in the region necessitates molecular data in ascertaining generic placements (Xu et al. [Bibr CR31][Bibr CR32]). For example, the transfer of *Chirita tamiana* B.L. Burtt to *Primulina* [i.e., *P. tamiana* (B.L. Burtt) Mich. Möller & A. Weber] made without support from molecular data by Weber et al. (2011) was recently challenged by chromosomal cytology and molecular data (Christie et al. 2012). Similarly, our ongoing molecular phylogenetic studies have also identified a number of misplacments in *Primulina* (Chung, unpubl. data).

In the course of a floristic survey in central Guangxi in 2007, a distinct species of Gesneriaceae with imparipinnate leaves was collected by the authors. After consulting national and local floras and the relevant literature (Wang et al. 1998; Li and Wang 2004; Wei 2010; Liu et al. 2011; Wang et al. 2011; Wu et al. 2012a; Xu et al. [Bibr CR31][Bibr CR32]) as well as herbarium specimens, we conclude that it represents a new species of *Primulina*, which is described and illustrated here. Chromosome count of the new species is in agreement with those reported for *Primulina* in Christie et al. (2012). Its generic placement is further confirmed by molecular and chromosome data.

## Methods

### Chromosome preparations

The plant for chromosome studies was collected from the type locality and cultivated in the experimental greenhouse of Academia Sinica, Taipei. A voucher specimen (*Ku et al. 2035*) has been deposited in HAST. Root tips were gathered and pretreated in 2 mM 8-hydroxyquinoline at 15–18°C for about 6 h and fixed overnight in an ethanol-acetic acid solution (3:1) below 4°C. The chromosomes were stained and macerated in 2% acetic orcein with 1 N hydrochloric acid (10:1). Classification of chromosome complement based on centromere position at mitotic metaphase followed Levan et al. (1964).

### Molecular methods

DNA sequences of the nuclear ribosomal internal transcribed spacers (ITS) and the chloroplast *trnL-F* intron spacer region were gathered using protocols outlined in Xu et al. (2012a). Because direct sequencing of the ITS PCR products of the new species resulted in DNA sequences with overlapping signals, molecular cloning was performed. Following the manufacturer’s protocol, the purified ITS templates were ligated to the pGEMT-T vector system (Promega, Madison, Wisconsin, USA) and subsequently transformed into competent cells (DH5α) to perform molecular cloning. After overnight culture at 37°C on the LB ampicillin/IPTG/X-gal selective plate, colonies carrying the ITS insert were identified by color (white) and further verified by PCR using the T7 and SP6 promoter primer pairs (Promega, Madison, Wisconsin, USA). Ten colonies with positive ITS insert were then transferred and grown in 2 μl LB medium at 37°C for 15 h. Plasmids were extracted using the Mini *Plus*™ Plasmid DNA Extraction System (Viogene, Taipei, Taiwan) and cycle-sequenced using the T7 and SP6 promoter primer pairs.

For phylogenetic analyses, matrices of Xu et al. (2012a) were adopted, keeping one accession for each species (Appendix 1) and ITS and *trnL*-*F* regions as separated matrices. *Primulina pinnata* (W.T. Wang) YinZ. Wang, which was not included in Xu et al. (2012a) because of the presence of an access of ambiguous sites in its ITS sequence in the GenBank (i.e., FJ501349), were added in current analyses to test for its putative relationships with the new species. The final matrix contained 24 species of *Primulina* with *Petrocodon dealbatus* Hance, *Pet. scopulorum* (Chun) YinZ. Wang, and *Didymocarpus podocarpus* C.B. Clarke chosen as outgroups based on recent phylogenetic analyses (Möller et al. 2011; Weber et al. 2011). The DNA sequences were aligned using the program MUSCLE implemented in the software MEGA5 (Tamura et al. 2011) with minor manual adjustments. Phylogenetic trees were reconstructed separately for ITS and *trnL-F* based on maximum parsimony (MP) and maximum likelihood (ML) criteria implemented in MEGA5. MP trees were searched using the Tree-Bisection-Reconnection (TBR) search option with the initial trees setting at 50, MP search level setting at 5, and maximum number of trees setting at 2000. Clade supports were calculated based on 100 bootstrap resamplings (parsimony bootstrap; PB). ML trees were reconstructed using the nearest-neighbor-interchange (NNI) method with all site used and the initial tree automatically selected under the model(s) selected by MEGA5. Clade supports of ML analysis were evaluated based on 100 bootstrap resamplings (likelihood bootstrap; LB).

## Results and discussion

### Taxonomic treatment

#### Primulina cardaminifolia

**Figure 1:**
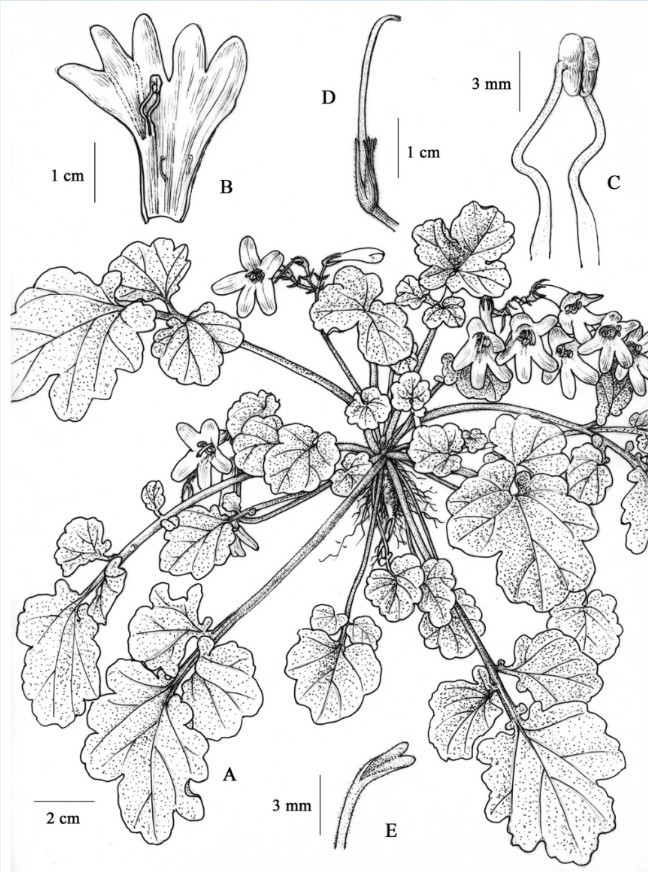
***Primulina cardaminifolia*** **Yan Liu &** **W.B. Xu. A**, Habit; **B**, Corolla opened to show stamens and staminodes; **C**, Stamens; **D**, Pistil and calyx; **E**, Stigma. (Drawn by W.H. Lin from the holotype).

**Figure 2:**
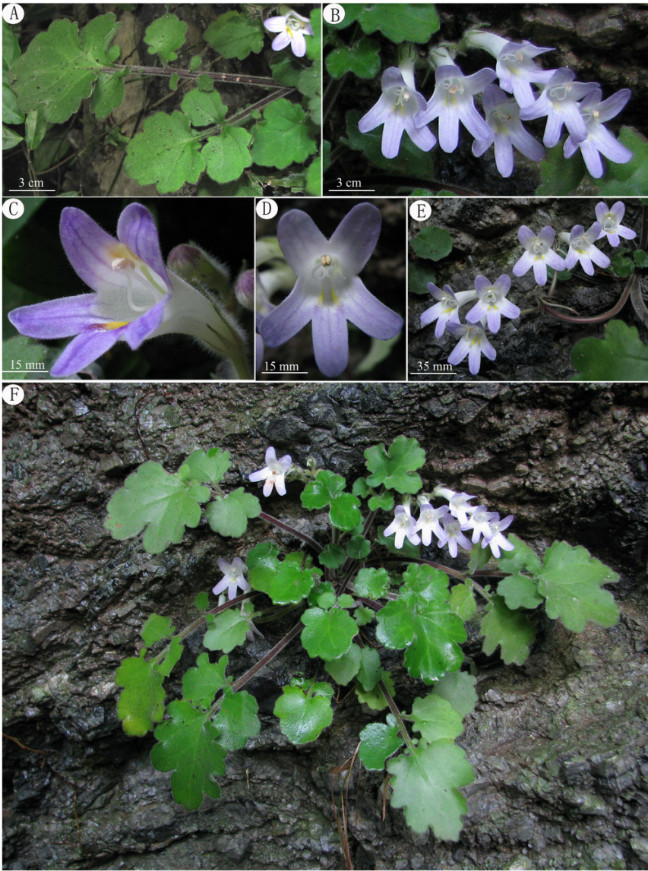
***Primulina cardaminifolia*** **Yan Liu &** **W.B. Xu. A**, Leaves; **B**, Flowers; **C**, Flower side view; **D**, Flower face view; **E**, Cyme; **F**, Habit.

Yan Liu & W.B. Xu, sp. nov.—TYPE: CHINA. Guangxi Zhuangzu Autonomous Region, Laibin Shi (City), Fenghuang Zhen (Township), alt. 280 m, on moist limestone rock face in a valley, 14 July 2008, *Wei-Bin Xu* & *Yan Liu 08050* (holotype: IBK; isotypes: HAST and PE). 碎米薺葉報春苣苔 Figures 1, 2.

#### Diagnosis

*Primulina cardaminifolia* Yan Liu & W.B. Xu resembles *Primulina pinnata* (W.T. Wang) YinZ. Wang in having imparipinnate leaves, but is clearly distinct from this species by the ovate-cordate terminal leaflet of 3–7 × 3–6.5 cm, 1 or 2 pairs of broadly ovate to sub-ovate lateral leaflets, 1–3-branched cymes with 3 to 10-flowers, and the entire-margined calyx lobes with acuminate apex.

#### Description

Herbs perennial. Rhizome subterete, 3–5 mm across. Leaves 5–7, in basal rosette, imparipinnate, 10–20 cm long, papery when dry; petiole subterete, 6–12.5 cm long, densely pubescent; terminal leaflet ovate-cordate, 3–7 × 3–6.5 cm, apex obtuse, base cordate, margin repand to irregularly pinnately lobed, densely pubescent on both surfaces; lateral leaflets 1 or 2 pairs, opposite or alternate, broadly ovate to rotund, 1–3 × 1–3 cm, margin repand to irregularly pinnately lobed, densely pubescent on both surfaces, petiolules short, 2–10 mm long. Cymes 2–4, axillary, 1–3-branched, 3–10-flowered; peduncle 4–8 cm long, 1–2 mm in diam., densely pubescent; bracts 2, opposite, linear-lanceolate, 7–8 × 2–3 mm, margin entire, pubescent; pedicel 4–7 mm long, densely pubescent. Calyx 5-parted to base, lobes lanceolate-linear, 8–12 × 2–3 mm, apex acuminate, outside pubescent, inside glabrous, margins entire. Corolla white to pale purple, 3.2–3.5 cm long, outside glandular-pubescent, inside sparsely puberulent, with 2 pale-yellow stripes; corolla tube 2.1–2.3 cm long, 8–12 mm in diam. at the mouth, 2.5–3 mm in diam. at the base; limb pale purple, distinctly 2-lipped; adaxial lip 2-parted to over the middle, lobes oblong, 8–10 × 6–7 mm; abaxial lip 3-lobed to over the middle, lobes oblong, 12–13 × 4–5 mm; stamens 2, adnate to 1.5 cm above the corolla tube base; filaments linear, ca. 1.2 cm long, geniculate above the base, sparsely glandular-puberulent; anthers ca. 3 mm long, ca. 1.5 mm wide, dorsifixed, glabrous; staminodes 2, ca. 5 mm long, apex capitate, glabrous, adnate to ca. 6 mm above the base of corolla tube. Disc ring-like, ca. 1 mm in height, margin repand, glabrous. Pistil 2.5–2.8 cm long, ovary 7–8 mm long, ca. 1.5 mm across, puberulent; style 1.5–1.8 cm long, ca. 0.6 mm across, puberulent; stigma obtrapeziform, ca. 2 mm long, apex 2-lobed. Capsules not seen.

#### Additional specimens examined

**CHINA**. Guangxi Zhuangzu Autonomous Region, Laibin Shi, Fenghuang Zhen, 3 July 2008, *Wei-Bin Xu* & *Yan Liu 08040* (IBK); same locality, 8 Sep 2008, *Wei-Bin Xu* & *Kuo-Fang Chung 08472* (IBK), *Shin-Ming Ku et al. 2035* (HAST); same locality, 28 June 2007, *Hong-Jin Wei* & *Wei-Bin Xu 07244* (IBK).

#### Ecology and distribution

**Figure 3:**
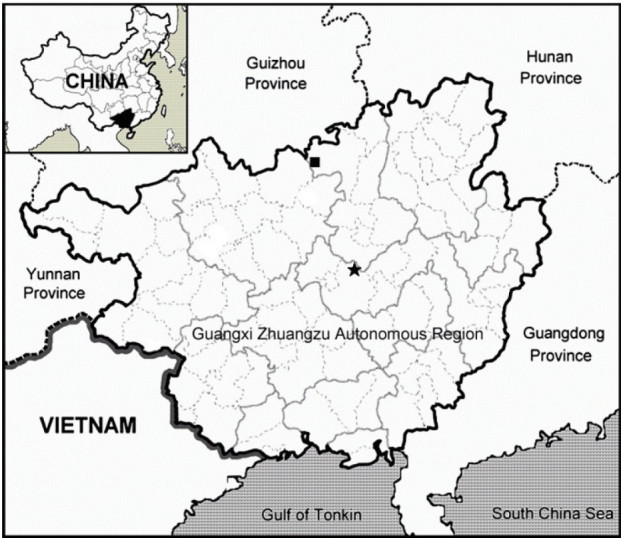
**Distribution of** ***Primulina cardaminifolia*** **(★) and** ***P. pinnata*** **(■) in Guangxi Zhuangzu Autonomous Region, China.**

*Primulina cardaminifolia* is extremely rare, currently known only from the type locality in Laibin Shi, Guangxi Zhuangzu Autonomous Region, China (Figure 3). It grows on a moist limestone rock face in a valley.

#### Phenology

Flowering from June to July; fruits not observed.

#### Etymology

The specific epithet is derived from the leaves resembling those of the genus *Cardamine* L. (Brassicaceae).

#### Notes

**Figure 4:**
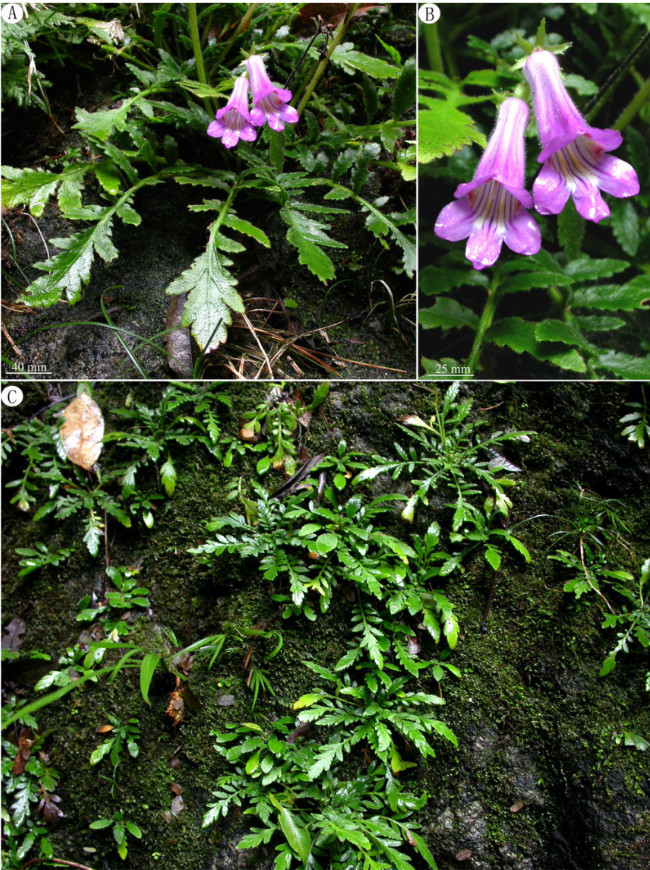
***Primulina pinnata*** **(W.T. Wang) YinZ. Wang. A**, Habit; **B**, Flowers; **C**, Habitat.

*Primulina cardaminifolia* resembles *Primulina pinnata* (W.T. Wang) YinZ. Wang (Figure 4), differing by the terminal leaflet being ovate-cordate, 3–7 × 3–6.5 cm (vs. oblong, 1.6–4.5 × 0.7–2.5 cm); the lateral leaflets 1 or 2 pairs, broadly ovate to rotund, 1–3 × 1–3 cm (vs. 3–5 pairs, oblong to oblong-lanceolate, 0.5–2.5 × 0.4–1.5 cm); cymes 1–3-branched, 3–10-flowered (vs. 1-branched, 1–3-flowered); calyx lobe with acuminate apex and entire margins (vs. acute to obtuse at apex and margins denticulate).

### Chromosome cytology

**Figure 5:**
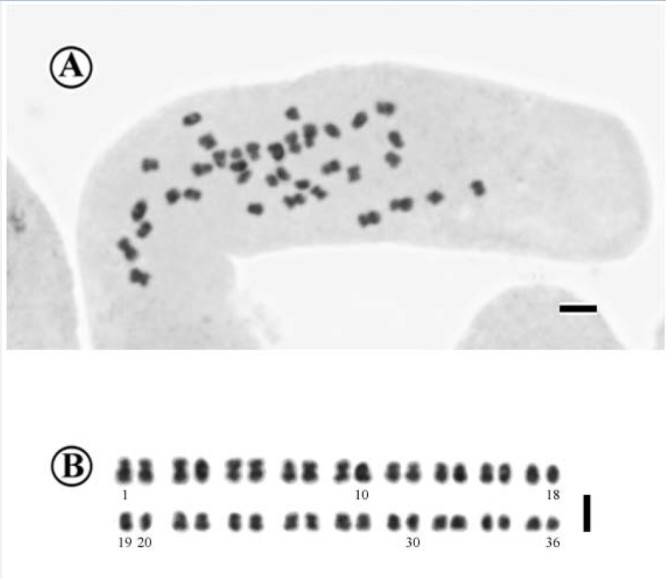
**Somatic chromosomes at metaphase of** ***Primulina cardaminifolia*** **(2** ***n*** **= 36, from** ***Ku et al. 2035*** **, HAST). A**, Microphotograph; **B**, Somatic chromosomes serially arranged by their chromosome length and the position of centromeres. Scale bars = 2 μm.

Somatic chromosomes at metaphase of *Primulina cardaminifolia* were determined to be 2*n* = 36 (Figure 5). The 36 chromosomes were small and gradually varied from ca. 0.6 μm to 1.3 μm in length. Most chromosomes had centromeres at median positions, while those of some of the shorter chromosomes could not be determined. Satellites were not observed.

In *Primulina*, chromosome numbers are uniformly diploid with 2*n* = 36 except for 2*n* = 32 in *P. tamiana* that was misplaced in the genus (Christie et al. 2012) and a polyploid with 2*n* = 72 in *P. longgangensis* (W.T. Wang) YinZ. Wang (Christie et al. 2012; Liu et al. 2012; Yang et al. 2012). Chromosomes of *Primulina* at somatic metaphase are generally small, ranging from 0.7 to 1.6 μm (Christie et al. 2012; Liu et al. 2012), to which *P. cardaminifolia* agrees (Figure 5). Our chromosome count of 2*n* = 36 in *P. cardaminifolia* agrees with basic chromosome number, *x* = 18, and supports its generic placement in the genus.

### Phylogenetic analyses

**Figure 6:**
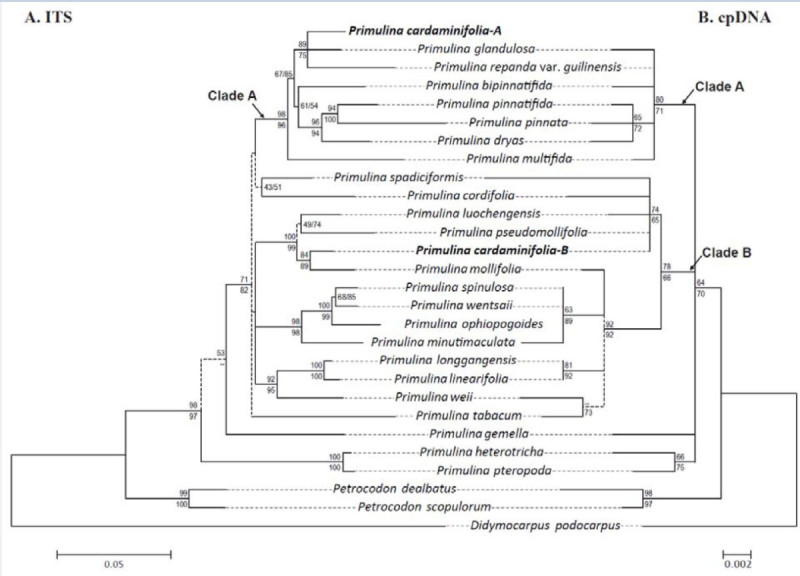
**Maximum likelihood phylogenies of A. ITS (left) and B.** ***trnL-F*** **intron spacer region (right) with clade supports (LB: upper; PB: lower).** Low support clades (LB and PB < 50%) are denoted by dash lines.

Results of molecular cloning of the ITS PCR product revealed two phylogenetically distinct ITS sequences (Figure 6A) with the length of 643 (*P. cardaminifolia*-A) and 634 (*P. cardaminifolia*-B) bp, respectively, while only one cpDNA sequence type was detected in the species. With the addition of these two ITS sequences, the ITS matrix contained 28 accessions of 675 aligned positions, of which 182 (26.96%) were parsimoniously informative. Based on the Kimura 2-parameter model using a discrete Gamma distribution (K2 + G) with 5 rate categories selected by the corrected Akaike Information Criterion (AICc) implemented in MEGA5, a single ML tree (log likelihood = −4114.9869) was recovered (Figure 6A). The MP analysis resulted in 6 equally parsimonious trees with 642 steps (CI = 0.70, RI = 0.64, RCI = 0.44). The topology of the strict consensus tree of the 6 equally parsimonious trees was congruent with the ML tree (not shown). The cpDNA matrix contained 27 accessions of 694 aligned positions, of which only 19 (1.3%) were parsimony informative. Based on the Hasegawa-Kishino-Yano (HKY) selected by the AICc implemented in MEGA5, a single ML tree (log likelihood = −1483.76) was obtained (Figure 6B). The MP analysis uncovered 23 equally parsimonious trees with 77 steps (CI = 0.97, RI = 0.97, RCI = 0.94). The topology of the strict consensus tree of the 270 equally parsimonious trees was largely consistent with the ML tree (not shown).

Both ITS and cpDNA dataset placed *P. cardaminifolia* in *Primulina* with high (LB = 98, PB = 97) and low (LB = 64, PB = 70) supports in ITS and cpDNA datasets, respectively (Figure 6), confirming its generic placement. Although the phylogenetic trees of the two datasets were not perfectly congruent with each other, especially in the deeper nodes, several well supported subclades were highly consistent between the two trees (Figure 6). Specifically, both datasets identified the clade (Clade A) composed of *P. glandulosa*, *P. repanda* var. *guilinensis*, *P. bipinnatifida*, *P. dryas*, *P. pinnatifida*, and *P. multifida*. In the ITS tree, the *P. cardaminifolia*-A sequence was also included in Clade A (Figure 6A). Other congruent clades included the clade consisting of *P. spinulosa*, *P. wentsaii*, *P. ophiopogoides*, and *P. minutimaculata*, the clade of *P. longgangensis* and *P. linearifolia*, and the clade of *P. heterotricha* and *P. pteropoda*.

**Figure 7:**
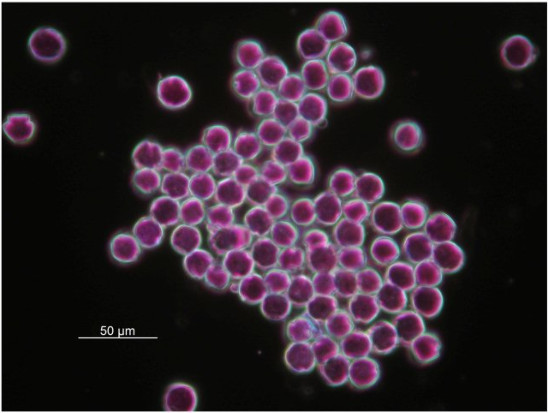
**Microphotographs of pollen of** ***Primulina cardaminfolia*** **, showing normal and fully stained pollen.**

The occurrence of intraindividual ITS polymorphism, or failure of concerted evolution among reiterated loci of ribosomal DNA arrays to nullify various rDNA repeats, could have resulted from hybridization, polyploidization, multiple nucelolar organizing regions on non-homologous chromosomes, rDNA pseudogenization, long generation time, loss of sexual recombination, or extensive introgression during domestication (Denduangboripant and Cronk 2000). The diploid chromosome number 2*n* = 36 and the presence of two phylogenetically distinct ITS sequence types raised the concern that the distinctive *P. cardaminifolia* might have been a hybrid (e.g., Peng and Chiang 2000), which has frequently been reported in Gesneriaceae (e.g., Puglisi et al. 2011). Assuming a maternal inheritance of its chloroplast genome, species in Clade B of the cpDNA tree would be the maternal parent, while Clade A could have been its paternal parent (Figure 6). Interestingly, most species in Clade A, including *P. glandulosa*, *P. bipinnatifida*, *P. pinnatifidai,* and *P. multifida*, are characterized by deeply lobed or pinnatified leaves that are otherwise unknown in *Primulina* and may have contributed to the unique imparipinnate leaves of *P. cardaminifolia* (Figures 1 & 2). Nevertheless, the species status of *P. cardaminifolia* was supported by a 100% pollen fertility suggested by the high level of stainable pollen (Figure 7) using the malachite green-acid fuchsin-orange G stain (Alexander 1969). Given its perfectly developed pollen, the cytological and molecular data instead could have suggested that *P. cardaminifolia* might be of homoploid hybrid origin, which was formed without changes in chromosome number (cf. Howarth and Baum 2005; Rieseberg and Willis 2007; Abbott et al. 2010). Further studies will be needed to test the probable hybrid and homoploid origin of the new species.

## Conclusion

All available data support the recognition of the new species *Primulina cardaminifolia*, which is described herein. The new species may have arisen via homoploid hybrid speciation. Its generic placement is confirmed by morphological, chromosomal, and molecular analyses.

## Appendix 1

GenBank accession numbers: Species: (ITS/*trnL-F*). *Primulina cardaminifolia* was collected from the type locality [*S.M. Ku 2035*(HAST)].

*Didymocarpus podocarpus* C.B. Clarke: (DQ912688/FJ501514); *Petrocodon dealbatus* Hance: (FJ501358/FJ501537); *Petrocodon scopulorum* (Chun) YinZ. Wang [=*Tengia scopulorum* Chun]: (GU350637/GU350669); *Primulina bipinnatifida* (W.T. Wang) Yin Z. Wang [=*Chiritopsis bipinnatifida* W.T. Wang]: (DQ872842/DQ872806); *Primulina cardaminifolia* Yan Liu & W.B. Xu: (ITS-A: JX506738, ITS-B: JX506739/JX506740); *Primulina cordifolia* (D. Fang & W.T. Wang) YinZ. Wang [=*Chiritopsis cordifolia* D. Fang & W.T. Wang]: (DQ872845/DQ872803); *Primulina dryas* (Dunn) Mich. Möller & A. Weber [=*Chirita sinensis* Lindl.]: (FJ501348/FJ501524); *Primulina gemella* (D. Wood) YinZ. Wang [=*Chirita gemella* D. Wood]: (FJ501345/FJ501523); *Primulina glandulosa* (D. Fang, L. Zeng & D.H. Qin) Yin Z. Wang [=*Chiritopsis glandulosa* D. Fang, L. Zeng & D.H. Qin]: (DQ872841/DQ872804); *Primulina heterotricha* (Merr.) YinZ. Wang [=*Chirita heterotricha* Merr.]: (DQ872826/DQ872816); *Primulina linearifolia* (W.T. Wang) YinZ. Wang [=*Chirita linearifolia* W.T. Wang]: (DQ872834/DQ872810); *Primulina longgangensis* (W.T. Wang) YinZ. Wang [=*Chirita longgangensis* W.T. Wang]: (FJ501347/AJ492290); *Primulina luochengensis* (Yan Liu & W.B. Xu) Mich. Möller & A. Weber [=*Wentsaiboea luochengensis* Yan Liu & W.B. Xu]: (HQ633046/HQ632949); *Primulina minutimaculata* (D. Fang & W.T. Wang) YinZ. Wang [=*Chirita minutimaculata* D. Fang & W.T. Wang]: (DQ872828/DQ872815); *Primulina multifida* B. Pan & K.F. Chung: (JX507031/JX506756); *Primulina ophiopogoides* (D. Fang & W.T. Wang) YinZ. Wang [=*Chirita ophiopogoides* D. Fang & W.T. Wang]: (DQ872829/DQ872814); *Primulina pinnata* (W.T. Wang) YinZ. Wang [*Chirita pinnata* W.T. Wang]: (FJ501349/FJ501526); *Primulina pinnatifida* (Hand.-Mazz.) Yin Z. Wang [=*Chirita pinnatifida* (Hand.-Mazz.) B.L. Burtt]: (FJ501350/FJ501527); *Primulina pseudomollifolia* W.B. Xu & Yan Liu: (JX506869/JX506759); *Primulina pteropoda* (W. T. Wang) Yan Liu [=*Chirita pteropoda* W.T. Wang]: (DQ872827/DQ872817); *Primulina repanda* var. *guilinensis* (W.T. Wang) Mich. Möller & A. Weber [=*Chiritopsis repanda* var. *guilinensis* W.T. Wang]: (DQ872846/DQ872808); *Primulina spadiciformis* (W.T. Wang) Mich. Möller & A. Weber [=*Chirita spadiciformis* W.T. Wang]: (FJ501346/AJ492291); *Primulina spinulosa* (D. Fang & W.T. Wang) YinZ. Wang [=*Chirita spinulosa* D. Fang & W.T. Wang]: (DQ872830/DQ872813); *Primulina tabacum* Hance: (FJ501352/AJ492300); *Primulina weii* Mich. Möller & A. Weber [=*Chirita mollifolia* D. Fang, Y.G. Wei & J. Murata]: (DQ872832/DQ872811); *Primulina wentsaii* (D. Fang & L. Zeng) YinZ. Wang [=*Chirita wentsaii* D. Fang & L. Zeng]: (DQ872831/DQ872812).

## Authors’ original submitted files for images

Below are the links to the authors’ original submitted files for images. Authors’ original file for figure 1 Authors’ original file for figure 2 Authors’ original file for figure 3 Authors’ original file for figure 4 Authors’ original file for figure 5 Authors’ original file for figure 6 Authors’ original file for figure 7 Authors’ original file for figure 8 Authors’ original file for figure 9
