# Supplementary figures and images for: Primulina cardaminifolia (Gesneriaceae), a rare new species from limestone areas in Guangxi, China
Source: Bot Stud. 2013 Aug 27;54:19. doi: 10.1186/1999-3110-54-19 (PMC5432832; doi:10.1186/1999-3110-54-19)

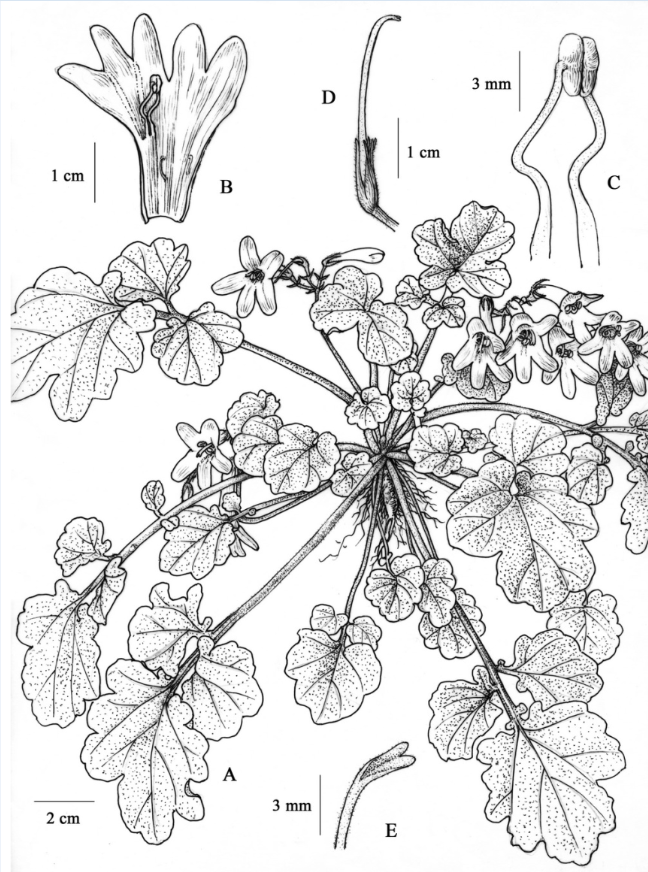

Supplement: Supplementary file 1 — Authors’ original file for figure 1 [file 40529_2012_18_MOESM1_ESM.png]

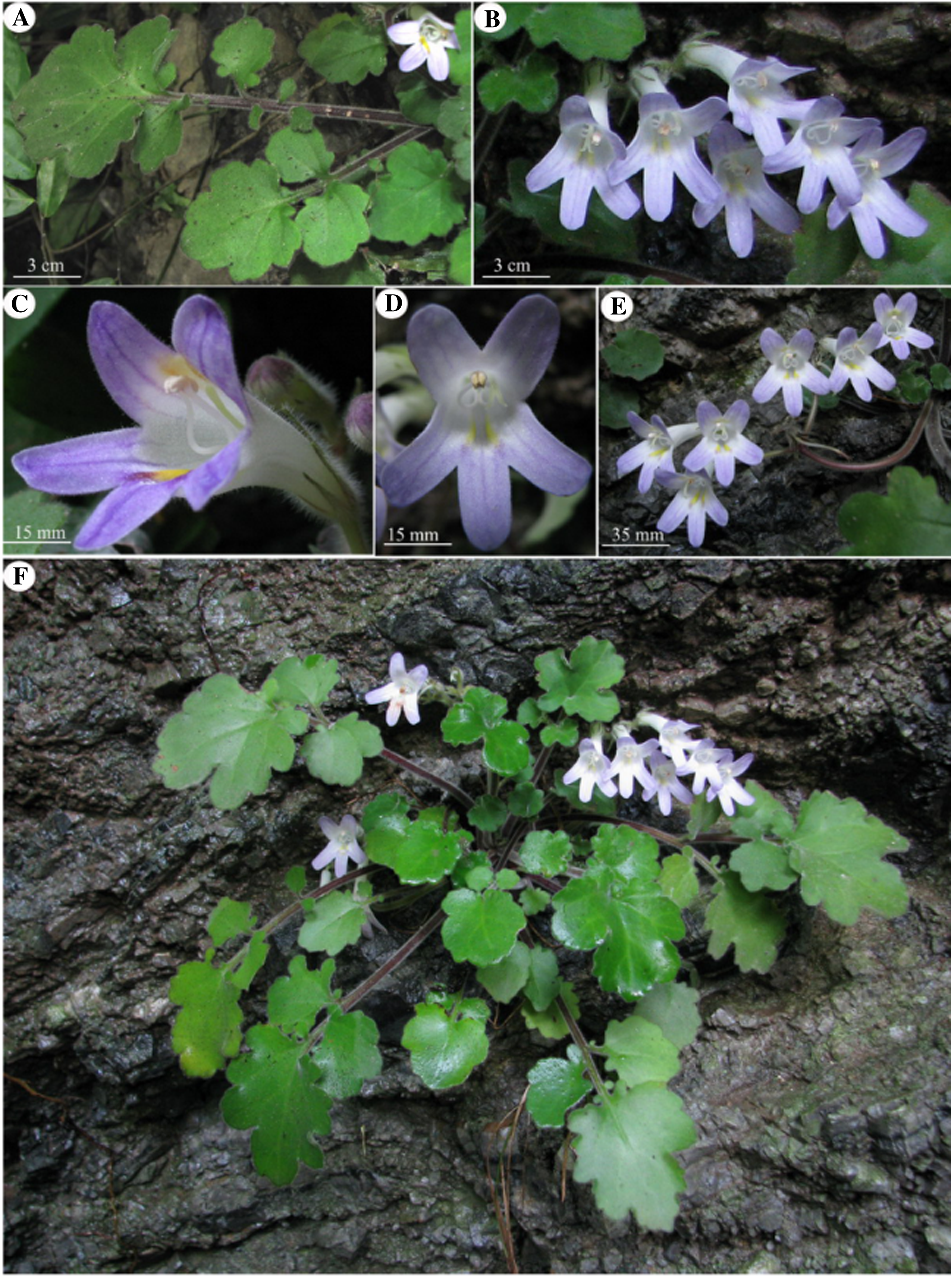

Supplement: Supplementary file 2 — Authors’ original file for figure 2 [file 40529_2012_18_MOESM2_ESM.tiff]

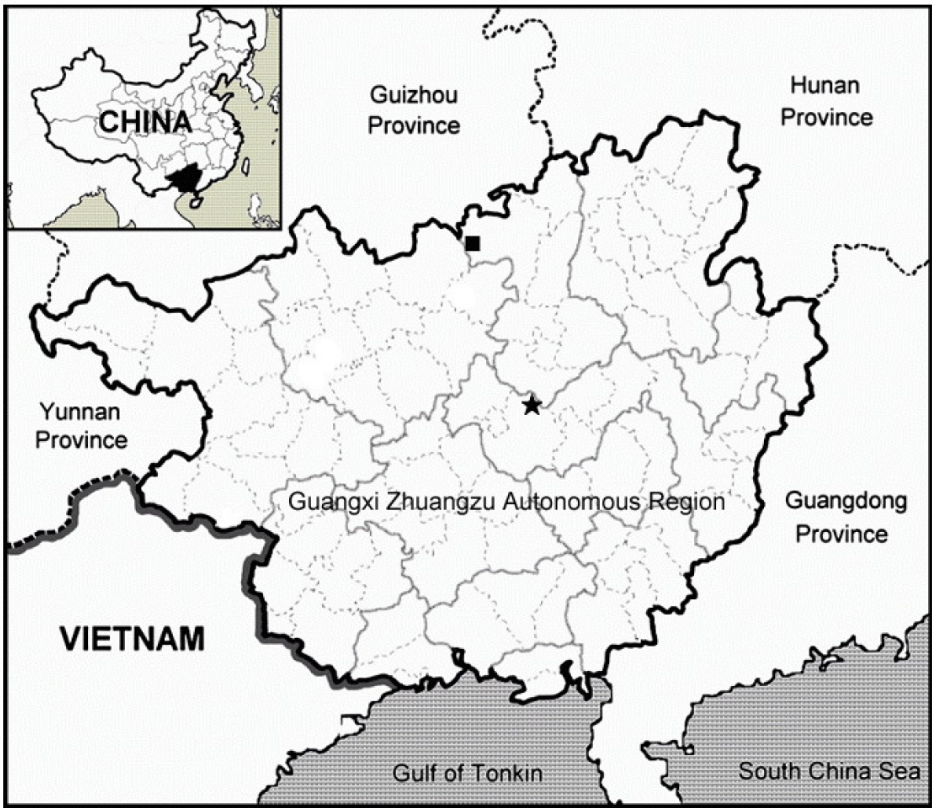

Supplement: Supplementary file 3 — Authors’ original file for figure 3 [file 40529_2012_18_MOESM3_ESM.png]

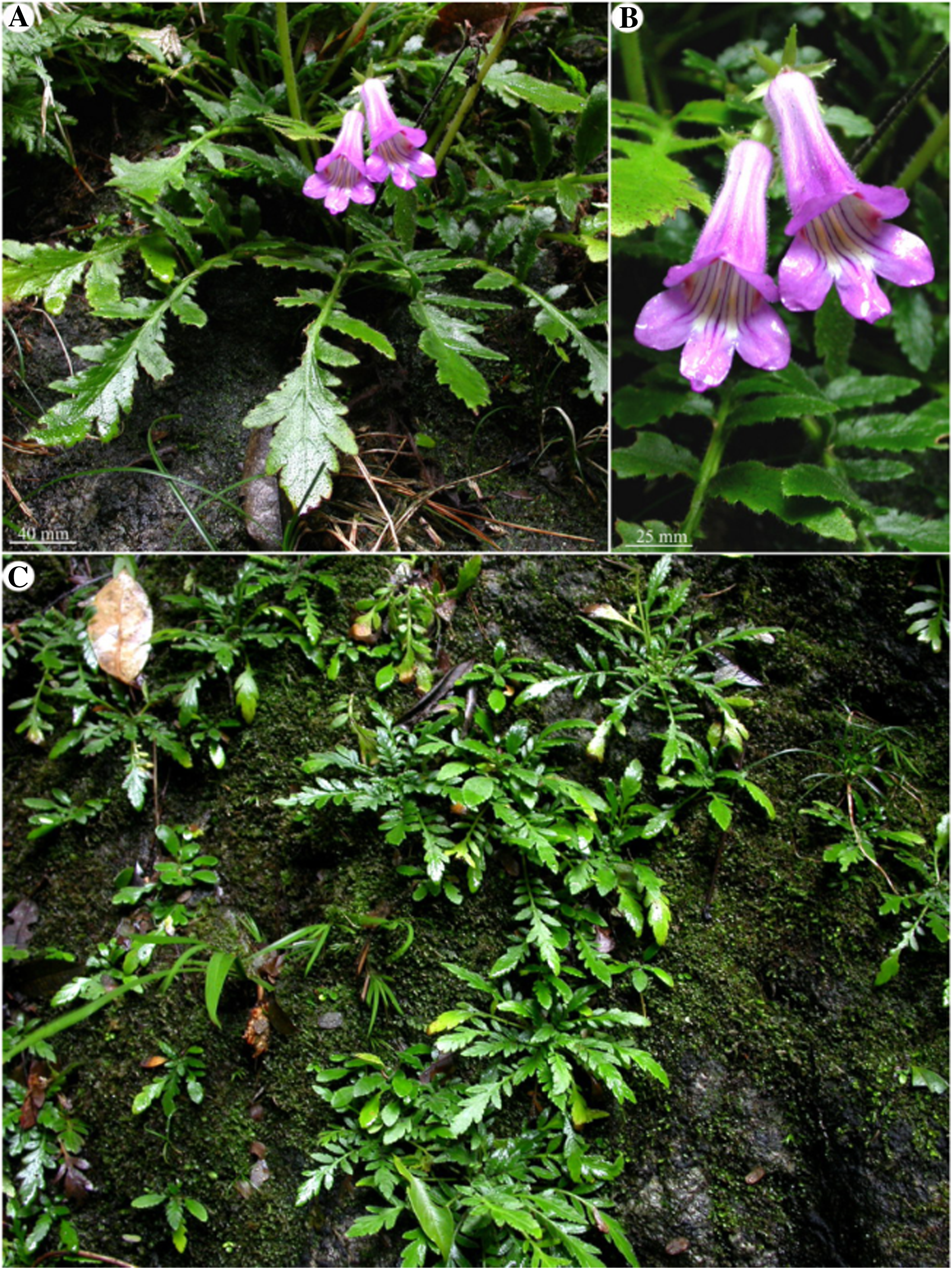

Supplement: Supplementary file 4 — Authors’ original file for figure 4 [file 40529_2012_18_MOESM4_ESM.tiff]

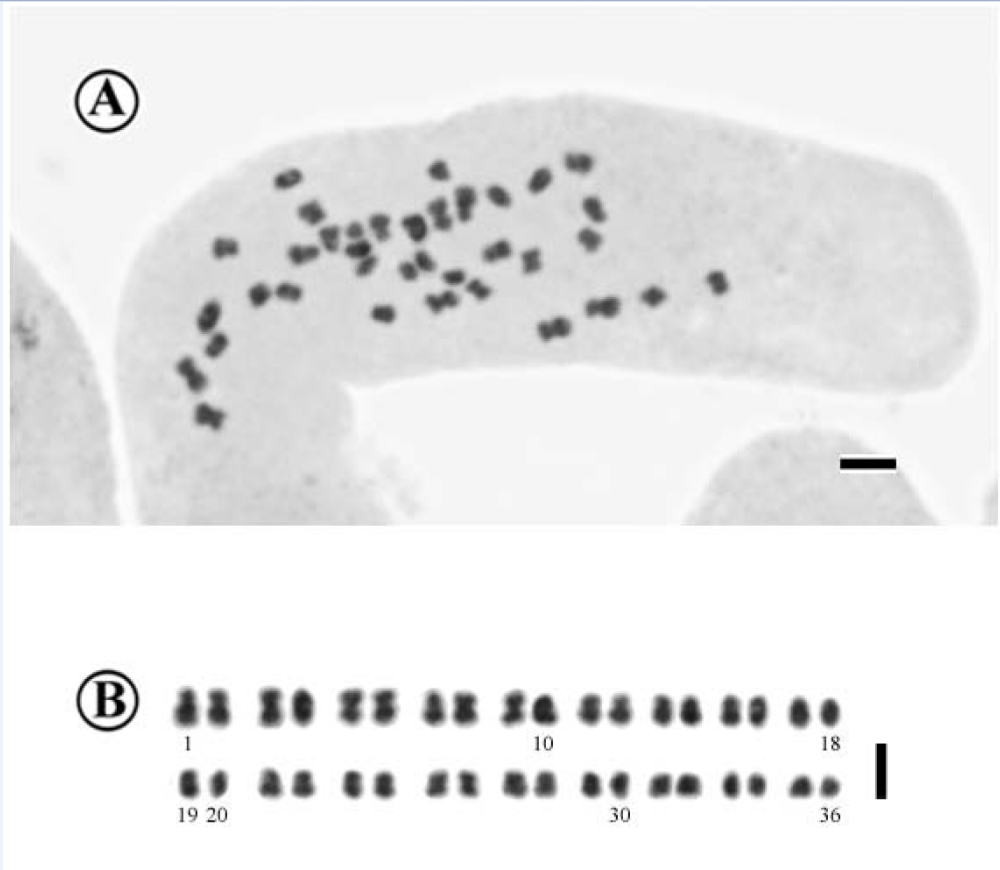

Supplement: Supplementary file 5 — Authors’ original file for figure 5 [file 40529_2012_18_MOESM5_ESM.png]

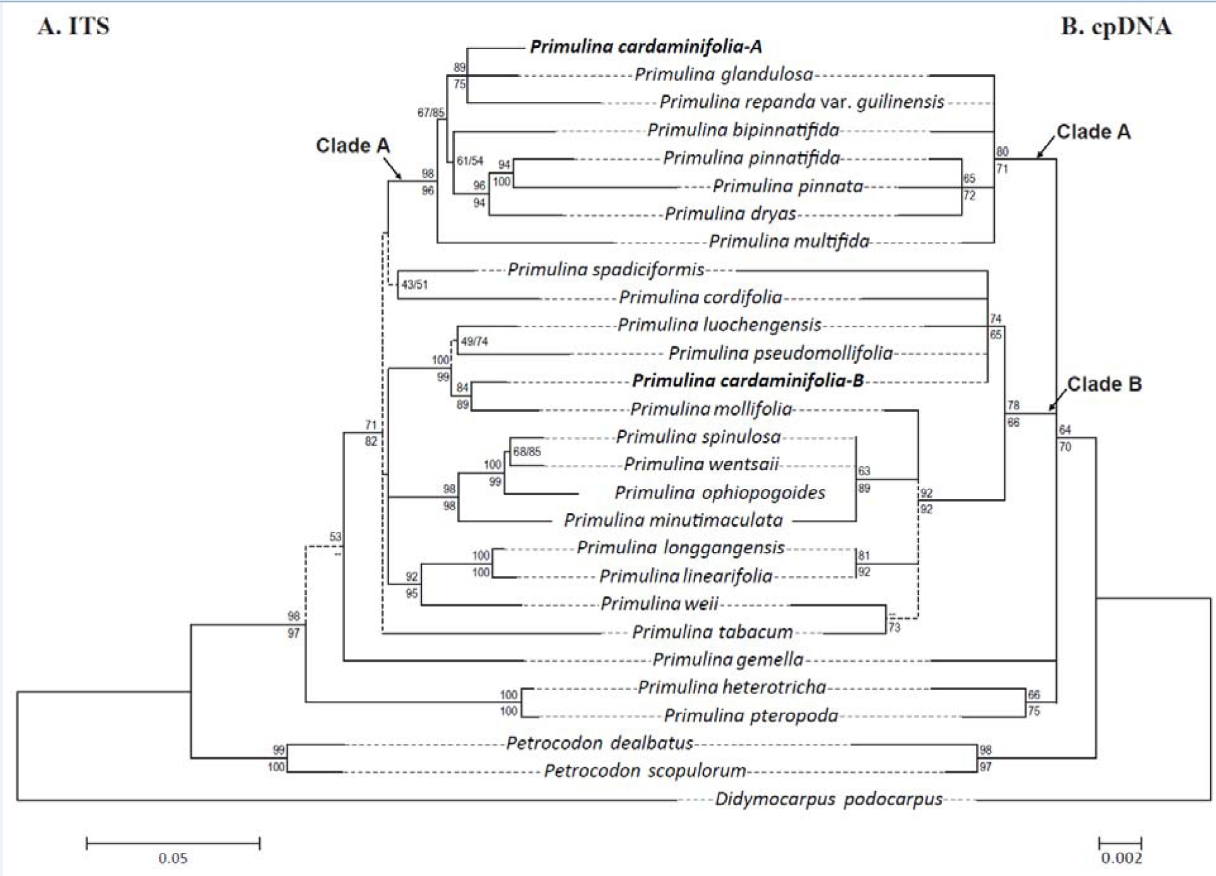

Supplement: Supplementary file 6 — Authors’ original file for figure 6 [file 40529_2012_18_MOESM6_ESM.png]

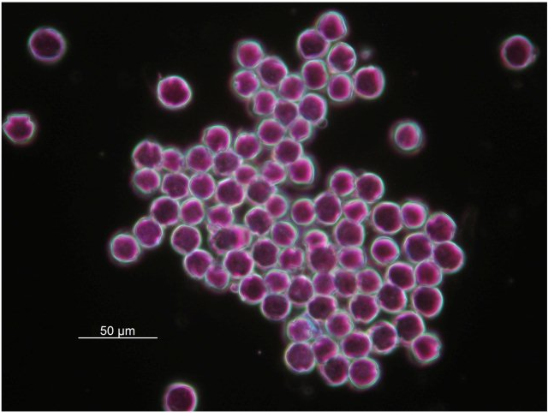

Supplement: Supplementary file 7 — Authors’ original file for figure 7 [file 40529_2012_18_MOESM7_ESM.png]

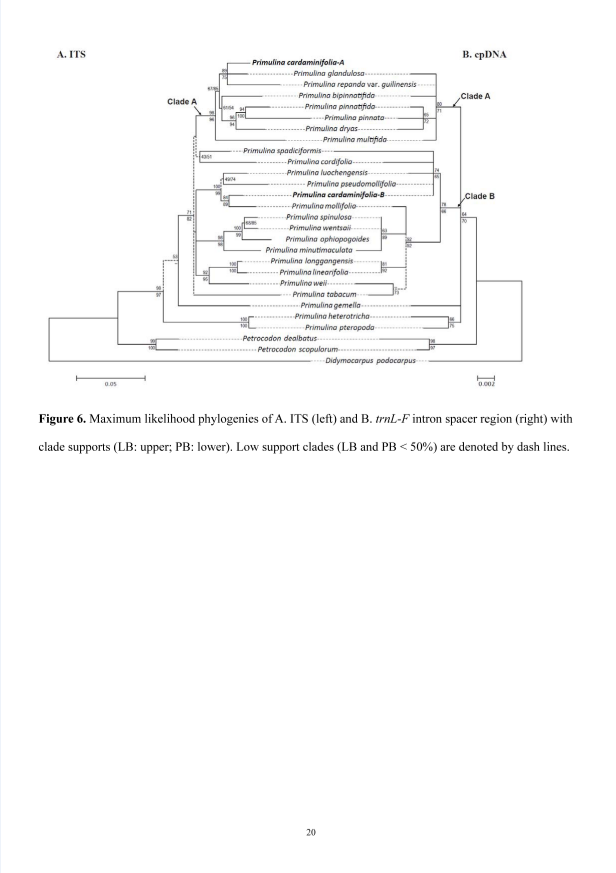

Supplement: Supplementary file 8 — Authors’ original file for figure 8 [file 40529_2012_18_MOESM8_ESM.png]

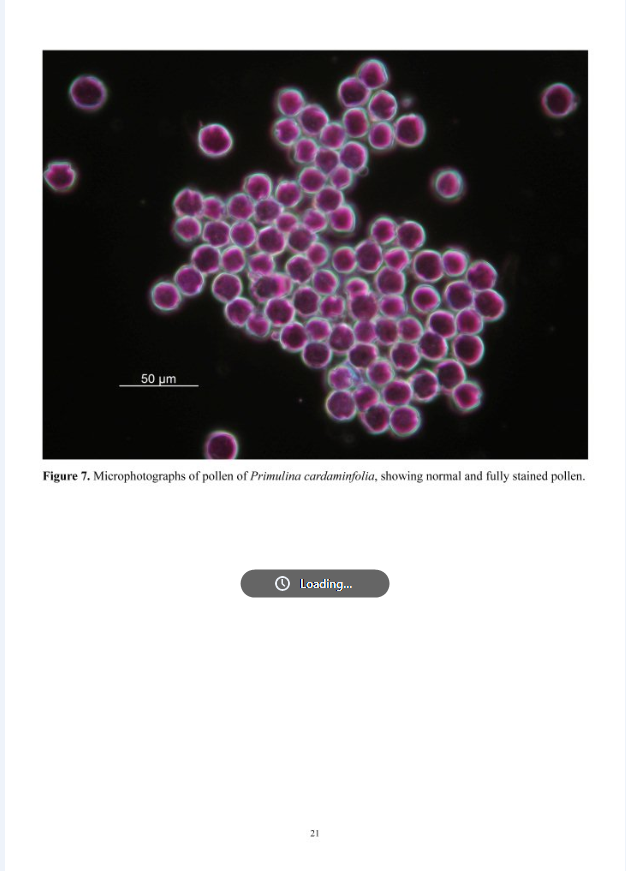

Supplement: Supplementary file 9 — Authors’ original file for figure 9 [file 40529_2012_18_MOESM9_ESM.png]
